# Development and psychometric validation of a multidimensional measure of embodied cognition in dance training

**DOI:** 10.3389/fpsyg.2026.1861862

**Published:** 2026-08-07

**Authors:** Yu Wang, Ziyu Zheng, Chenlu Ran, Qing Liu

**Affiliations:** 1 School of Music and Dance, Heze University, Heze, Shandong, China; 2 Mental Health Center, Heze University, Heze, Shandong, China

**Keywords:** dance training, embodied cognition, factor analysis, measurement invariance, psychometric validation, scale development

## Abstract

**Background:**

Embodied cognition has been increasingly recognized as an important mechanism linking bodily experience and psychological adaptation, yet few instruments capture its multidimensional characteristics in dance training contexts.

**Objective:**

The present study aimed to develop and validate a measure of embodied cognition for dance training.

**Methods:**

A total of 439 undergraduate dance students participated in a two-stage cross-validation design. Exploratory factor analysis was conducted in a pilot sample (*n = 136*) to identify the underlying factor structure, and confirmatory factor analysis was performed in an independent sample (*n = 303*) to test the model. Reliability and validity were evaluated using internal consistency, composite reliability, average variance extracted, and discriminant validity indices.

**Results:**

The results supported a five-factor structure comprising motor control, positive body self-image, cognitive flexibility, mind-body integration, and rigid response/negative experience. The scale showed acceptable model fit and satisfactory reliability and validity.

**Conclusion:**

These findings provide preliminary support for a multidimensional measure of embodied cognition in dance training and offer a context-sensitive tool for future research on embodied processes in highly trained physical performers.

## 1 Introduction

Embodied cognition has emerged as a major theoretical shift in 21st-century cognitive science, evolving from conceptual discourse to an empirically operationalized research framework. In contrast to traditional disembodied approaches, which regard the body as a passive carrier of cognitive processing, contemporary embodied cognition theories emphasize that cognitive activity is grounded in bodily experience, bodily structure, and the dynamic coupling between organism and environment (Barsalou, 2020; Shapiro, 2019; Wilson, 2002). From this perspective, embodied cognition is shaped by the dynamic interplay of perceptual, affective, and motor experiences, as well as the physical competencies that enable situated action (Barsalou, 2020; Wilson, 2002).

From an enactive perspective, cognition is not an internal representation of the external world but rather an emergent property of the structural coupling between body and environment (Varela et al., 1991; Thompson, 2007). Body schema theory further underscores the constitutive role of the lived body in cognitive processes from a phenomenological standpoint (Gallagher, 2005).

In recent years, embodied cognition has received increasing attention in educational and performing arts contexts. Empirical evidence suggests that sensorimotor coupling processes contribute to the development of cognitive flexibility and self-efficacy (Koch et al., 2014; Glenberg, 2010). In highly embodied training environments, individuals must integrate bodily awareness, motor control, and emotional regulation processes simultaneously (Mehling et al., 2011). Neuroimaging research indicates that long-term dance training enhances functional connectivity within the action observation–execution network (including the mirror neuron system), the basal ganglia–cerebellar circuits, and the interactions between the default mode network (internally oriented cognition) and the salience network (attentional switching), supporting stable patterns of cognitive–motor integration (Calvo-Merino et al., 2005; Cox et al., 2024; Vander Elst et al., 2023). However, existing research has largely remained at the level of theoretical elaboration or neural mechanisms. Multidimensional measurement instruments tailored to highly trained embodied populations remain limited, making it difficult to systematically assess key constructs such as motor control, cognitive flexibility, and body self-representation within professional dance contexts.

Currently, widely used instruments such as the Multidimensional Assessment of Interoceptive Awareness (MAIA; Mehling et al., 2012) and body awareness scales (Price and Thompson, 2007) were primarily developed for clinical or general populations. Their measurement focus centers on static bodily awareness and body-related anxiety. In contrast, measurement tools capturing motor control capacity, positive body self-image, and situational regulation ability within high-intensity physical training contexts remain insufficient (Nordin-Bates et al., 2012; Schaefer et al., 2018). During embodied training, embodied cognition manifests not only as static awareness but also as dynamic regulation during movement execution, including cognitive flexibility in improvisation and body–cognition coordination in motor sequence learning (Glenberg, 2010). Theoretical accounts argue that movement itself constitutes cognitive processing rather than merely expressing pre-existing mental representations (Sheets-Johnstone, 1999; Clark, 2008). Nevertheless, existing instruments rarely differentiate between motor control capacity and cognitive regulatory capacity, nor do they independently assess their integrative mechanisms.

Moreover, high-intensity physical training involves distinctive stress contexts, including bodily frustration during technical plateaus, experiences of mind–body separation in stage performance, and long-term bodily strain due to repetitive practice (Aujla et al., 2021). Empirical findings indicate that dancers report significantly higher levels of social physique anxiety compared to non-dancers (Quested and Duda, 2009). However, existing scales lack sensitivity to constructs such as rigid response patterns and negative embodied experiences, making it difficult to distinguish adaptive bodily awareness from maladaptive hyper-monitoring. Although the MAIA proposes a multidimensional structure, its structural stability in athletic and highly trained populations remains debated. Studies have identified psychometric concerns in sport samples, including imbalanced factor loadings and inconsistencies in reverse-scored items (Satterly et al., 2022). Additionally, the scale does not explicitly capture dimensions such as mind–body disconnection or dissociative experiences, limiting its capacity to assess integrative states under high-pressure conditions. Overall, existing research frequently conceptualizes embodied cognition as a unitary construct, overlooking its dimensional heterogeneity in physically intensive training contexts.

Systematic physical training substantially enhances the precision of interoceptive and proprioceptive signal processing. Research demonstrates that professional dancers exhibit significantly higher interoceptive accuracy in heartbeat tracking tasks compared to non-dancers, and such accuracy positively correlates with years of training (Christensen et al., 2017). Furthermore, dancers outperform control groups in spatial localization tasks relying on proprioceptive cues (Jola et al., 2011), indicating superior integration of internal bodily feedback. This dual sensitivity supports refined motor control and emotional expression (Mehling et al., 2012; Koch et al., 2014). Long-term training also strengthens the predictive and regulatory capacities of the body as an instrument of action. Neurocomputational research highlights the role of cerebellar–premotor closed-loop circuits in action prediction and optimization (Caligiore et al., 2013), contributing to the development of higher-order agency.

In terms of situational adaptation, individuals achieve dynamic motor regulation and generative adaptation through continuous interaction with the environment (Ravn and Hansen, 2013). Such adaptive capacity involves real-time perception of action possibilities and energy transformation (Ravn, 2017), reflecting the generative characteristics of body–environment coupling. Complex movement decisions and rhythmic modulation further require heightened situational sensitivity (Calvo-Merino et al., 2005). In addition, physical training strengthens processes of emotional embodiment. Evidence indicates that professional dancers exhibit greater physiological differentiation during emotional action processing (Christensen et al., 2016). Movement execution and observation have been shown to modulate emotional states effectively (Shafir et al., 2015), and bodily experience shapes emotional awareness and expression through sensorimotor coupling mechanisms (Koch et al., 2014). Therefore, in physically intensive training contexts, embodied cognition manifests as a multidimensional synergy of motor control, situational adaptation, and emotional integration.

Based on the aforementioned theoretical and empirical background, the present study aims to: (1) construct and refine an item pool of embodied cognition tailored to highly physically trained populations, and determine the preliminary factor structure through expert review and exploratory factor analysis; and (2) conduct confirmatory factor analysis in an independent sample to examine the scale’s structural validity, reliability, and related validity indicators, thereby establishing a multidimensional measure of embodied cognition with sound psychometric properties.

## 2 Materials and methods

### 2.1 Participants

A two-stage cross-validation sampling design was employed in this study. During the pilot phase, undergraduate dance students were recruited through convenience sampling and snowball sampling from a dance academy at a university in Shandong Province and from dance departments of comprehensive universities. A total of 148 questionnaires were distributed, of which 12 were excluded due to incomplete responses (*n* = 8), straight-line responding (*n* = 3), or inconsistent demographic information (*n* = 1), yielding 136 valid responses (effective response rate = 91.9%). The demographic characteristics of the pilot sample were as follows: 25.0% male (*n* = 34) and 75.0% female (*n* = 102); grade distribution: first year (30.9%), second year (27.9%), third year (25.7%), and fourth year (15.4%); primary dance specialization: Chinese classical dance (41.2%), folk dance (32.4%), ballet (16.2%), and modern dance (10.2%).

During the formal survey phase, 303 valid undergraduate dance majors from a university in Shandong Province were included. The demographic characteristics were as follows: 26.7% male (*n* = 81) and 73.3% female (*n* = 222); grade distribution: first year (51.5%), second year (24.1%), third year (14.5%), and fourth year (9.9%); primary dance specialization: artistic performance (75.9%) and competitive dance (24.1%); mean years of training = 8.4 years (SD = 3.2).

Based on the effect size observed in the pilot phase (r ≈ 0.30), an a priori power analysis using G*Power 3.1 indicated that a minimum sample size of 250 was required to detect a medium effect size (f^2^ = 0.15) with power = 0.80 at α = 0.05. The formal survey sample size (*N* = 303) met this requirement, ensuring adequate statistical power.

### 2.2 Instrument development

Scale development followed rigorous psychometric procedures and consisted of four stages.

Theoretical Construction and Literature Review: Grounded in generative embodied cognition theory, a multidimensional structure of embodied cognition in dance contexts was constructed by integrating body-self theory, perceptual control theory, and cognitive flexibility theory. Relevant subscales from the MAIA (Attention Regulation and Emotional Awareness), the situational adaptation dimension from cognitive flexibility scales, and related items from body self-relation scales were referenced. Contextualized items were developed based on motor control theory. All items were rewritten to reflect dance-specific contexts. An initial pool of 40 items was generated.

Expert content validity evaluation: Following the triangulation principle, five experts were selected, including two professors of dance education, two experts in sport psychology, and one expert in psychometrics, all holding associate senior titles or above. A modified Lynn three-point scale (1 = highly relevant/clear/appropriate; 0 = questionable; −1 = not relevant) was used for evaluation. The item-level content validity index (I-CVI) and scale-level content validity index (S-CVI/Ave) were calculated.

Results showed Kendall’s W = 0.84 (χ^2^ = 378.25, df = 89, *p* < 0.001), indicating a high level of agreement among experts. All 40 initial items achieved I-CVI ≥ 0.80, and S-CVI/Ave = 0.98. No item fell below the recommended threshold (0.78). Detailed expert review ratings for the 40 initial items are provided in Supplementary Tables 1, 2. The final ECAS-27 questionnaire is provided in Supplementary material.

Cognitive Interviews: Six dance majors (three males and three females), representing different grades and dance specializations, participated in semi-structured interviews to assess item clarity and contextual appropriateness. Item wording was refined accordingly to address ambiguities.

Pilot testing and item screening: Item analysis was conducted based on the pilot sample (*N* = 136). According to the critical ratio (CITC < 0.30) and theoretical appropriateness, five items were initially deleted. Regarding the F5 (mind–body integration) dimension: exploratory factor analysis in the pilot phase indicated that the original five items all loaded on the intended factor. However, EC-M4 (“the body’s needs should take priority”) and EC-M5 (“I feel proud of my physical abilities”) showed conceptual overlap, both involving evaluation of bodily competence. To avoid redundancy and enhance factorial purity, the three most representative items (EC-M1 to EC-M3) were retained, corresponding to the core elements of action–attention synchrony, focused mind–body fusion, and awareness of present bodily needs. Ultimately, 27 items were retained for the formal survey. The F5 (mind–body integration) dimension was considered irreplaceable by experts and interview participants for representing mind–body integration and dissociative experience in dance training. Although the number of usable items was limited (3 items), the dimension was retained to cover the dance-specific construct of “mind–body disconnection” and to meet the basic identification requirements of reflective measurement models. All three items were reverse-scored, measuring dissociative experience. After reverse coding, higher scores indicated higher levels of mind–body integration.

The final 27-item ECAS uses a 5-point Likert scale ranging from 1 (strongly disagree) to 5 (strongly agree). Sample items include: “I can control the force, speed, and direction of my movements precisely” (F1, Motor Control); “I feel satisfied with my body shape” (F3, Positive Body Self-Image); and “When I am focused, my mind and body feel completely disconnected” (F5, Mind–Body Integration, reverse-scored).

### 2.3 Procedure

Data collection followed standardized procedures. The researchers obtained course schedules from the academic affairs office of the dance school and distributed paper questionnaires during class breaks. Independent response spaces were arranged to minimize social desirability effects.

All participants signed informed consent forms and were clearly informed of the study purpose, confidentiality principles, and voluntary withdrawal rights. The study was approved by the Ethics Committee of Heze University (Approval No. HZU20250515) and adhered to the ethical principles of the Declaration of Helsinki.

Questionnaire completion time was controlled within 10–15 min. Questionnaires were collected on-site and checked immediately for completeness.

In the formal survey phase, demographic variables (gender, grade, primary dance specialization, years of training) and criterion variables (psychological resilience and years of dance training) were simultaneously collected for criterion-related validity testing.

### 2.4 Data analysis

Statistical analyses were conducted using SPSS 26.0 and AMOS 26.0. During data preprocessing, all negatively worded items were reverse-coded to ensure consistent scoring direction across dimensions (DeVellis, 2017; Netemeyer et al., 2003).

Exploratory factor analysis: In the pilot phase, principal component analysis (PCA) was used for factor extraction. PCA was selected over common factor extraction methods [e.g., Principal Axis Factoring (PAF) or Maximum Likelihood (ML)] because the primary goal was data reduction and item retention decisions rather than latent variable modeling. PCA is robust to distributional violations and computationally stable for pilot-phase item screening, consistent with scale development best practices (Costello and Osborne, 2005; Fabrigar et al., 1999). However, we acknowledge that PAF or ML may provide more accurate communality estimates when strong common factors are expected. As a sensitivity check, we re-ran the EFA using PAF with Promax rotation; the five-factor structure and item loadings were highly consistent with the PCA solution (congruence coefficients > 0.95 for all factors), supporting the stability of the identified structure across extraction methods. The number of factors was determined using the Kaiser criterion and parallel analysis (Hayton et al., 2004). Promax rotation was applied, allowing correlations among factors (Fabrigar et al., 1999). Item retention criteria were as follows: primary factor loading greater than 0.40; cross-loading difference less than 0.25 or secondary loading less than 0.30; communalities (h^2^) greater than 0.30 (Costello and Osborne, 2005; Worthington and Whittaker, 2006); and theoretical interpretability of item content. For the F5 dimension, given its theoretical specificity and small number of items (3 items), particular attention was paid during EFA to its discriminant validity relative to F3 (positive body self-image), in order to avoid cross-dimensional loadings caused by content overlap.

Confirmatory factor analysis: In the formal survey phase, three competing models were constructed and compared: a strictly unidimensional model (all 27 items loading on a single factor), the first-order correlated five-factor model, and the second-order single-factor model (Brown, 2015; Kline, 2016). Maximum likelihood (ML) estimation was used for parameter estimation (Bollen, 1989). Model fit was evaluated using χ^2^/df, root mean square error of approximation (RMSEA), comparative fit index (CFI), Tucker–Lewis index (TLI), and standardized root mean square residual (SRMR) (Hu and Bentler, 1999). Given the sensitivity of fit indices to model complexity, traditional cutoff values were used as reference criteria, combined with considerations of model complexity and parameter plausibility (Goretzko et al., 2023; Marsh et al., 2004). Model comparison followed the criterion proposed by Chen (2007), whereby ΔCFI < 0.01 was considered indicative of negligible differences between models.

Reliability testing: Cronbach’s α and composite reliability (CR) were calculated (Fornell and Larcker, 1981). For F5 (3 items), given the limited number of items, CR was used as the primary reference indicator, with α serving as a supplementary reference (Peterson and Kim, 2013; Cho and Kim, 2015).

Validity testing: Convergent validity was assessed using average variance extracted (AVE), with AVE greater than 0.50 as the criterion (Fornell and Larcker, 1981; Hair et al., 2019). Discriminant validity was examined using both the Fornell–Larcker criterion and the heterotrait–monotrait ratio (HTMT) (Henseler et al., 2015).

Common Method Bias: Because all data were collected via self-report questionnaires, Harman’s single-factor test was conducted to assess common method bias (Podsakoff and Organ, 1986). Unrotated exploratory factor analysis indicated that the first factor accounted for 28.3% of the variance, suggesting that common method bias did not pose a serious threat to the validity of the results (Podsakoff et al., 2003; Kline, 2016). However, we acknowledge that Harman’s single-factor test has known limitations, including low sensitivity to detect method effects when multiple substantive factors exist (Podsakoff et al., 2003). As a complementary approach, we examined the correlation matrix for extremely high correlations (r > 0.90) between conceptually distinct variables, finding none. Future research should consider marker variable techniques or latent method factor modeling to more rigorously control for common method variance.

## 3 Results

### 3.1 Item analysis

Based on the pilot sample (*N* = 136), item analysis was conducted for the Embodied Cognition Assessment Scale (ECAS). Table 1 presents the descriptive statistics and discrimination indices for the final 27 items retained for the formal survey (F1: 7 items; F2: 5 items; F3: 6 items; F4: 6 items; F5: 3 items).

**TABLE 1:** Descriptive statistics and item analysis of the pilot-stage candidate items (*N* = 136).

Item	Item description (abbreviated)	M	SD	Skewness	Kurtosis	CITC	α if deleted
EC-A1	Good coordination of hands/limbs	4.12	0.85	−0.42	0.21	0.68	0.87
EC-A2	Control of force, speed, direction	4.05	0.88	−0.38	0.15	0.71	0.87
EC-A3	Stable mastery of new movements	3.98	0.92	−0.35	0.08	0.69	0.87
EC-A4	Regular weekly training	4.25	1.12	−0.85	0.62	0.31	0.90*
EC-A5	Persist despite fatigue	3.76	1.05	−0.28	−0.15	0.65	0.88
EC-A6	Endurance in moderate-intensity activity	3.82	1.08	−0.32	−0.08	0.64	0.88
EC-A7	Rapid adjustment to venue changes	3.95	0.98	−0.41	0.12	0.58	0.89
EC-C1	Generate multiple options before decisions	3.89	0.94	−0.36	0.18	0.62	0.76
EC-C2	Verify information sources	4.02	0.87	−0.45	0.25	0.58	0.77
EC-C3	Incorporate others’ perspectives	4.15	0.82	−0.52	0.38	0.65	0.75
EC-C4	Weigh pros and cons before decisions	3.88	0.91	−0.33	0.14	0.67	0.75
EC-C5	Adjust quickly after plan failure	3.72	0.96	−0.25	0.05	0.51	0.79
EC-C6	View issues from different angles	3.95	0.89	−0.38	0.22	0.63	0.76
EC-I1	Satisfied with body shape	3.65	1.12	−0.18	−0.42	0.72	0.88
EC-I2	Satisfied with movement and appearance	3.58	1.15	−0.12	−0.48	0.78	0.87
EC-I3	Proud of my body	3.62	1.18	−0.15	−0.45	0.75	0.88
EC-I4	My body brings me joy	4.08	0.95	−0.48	0.32	0.68	0.89
EC-I5	My body is flexible	3.85	1.02	−0.35	−0.12	0.58	0.9
EC-I6	My whole body feels powerful	3.78	1.08	−0.28	−0.22	0.59	0.9
EC-M1	When I am focused, my mind and body feel completely disconnected	3.52	1.08	−0.15	−0.35	0.48	0.59*
EC-M2	During performance, I feel like my body is moving on its own, separate from my mind	3.45	1.12	−0.08	−0.48	0.65	0.52*
EC-M3	I often lose awareness of my body’s present needs during training	3.62	1.05	−0.22	−0.28	0.71	0.48*
EC-B1	Anxiety or fear about the body	2.85	1.25	0.42	−0.28	0.68	0.72
EC-B2	Concern about appearance pressure	3.12	1.28	0.28	−0.45	0.52	0.78
EC-B3	Panic when situations change	2.95	1.22	0.38	−0.32	0.74	0.71
EC-B4	Give up easily when activity becomes difficult	2.78	1.18	0.48	−0.22	0.69	0.73
EC-B5	Difficulty adapting to unexpected changes	2.92	1.2	0.35	−0.3	0.7	0.74

F5 (EC-M1–EC-M3) consists of reverse-scored items. The descriptive statistics reported here reflect the original (pre-reverse-coded) scores. *Indicates that deletion of the item would increase the Cronbach’s α of the corresponding dimension; however, the item was retained based on content validity and theoretical justification.

Regarding distributional characteristics, the absolute values of skewness for all items were less than 2, and the absolute values of kurtosis were less than 7, meeting the empirical criteria for multivariate normality (Kline, 2016). These results satisfied the statistical assumptions required for subsequent factor analyses.

In terms of item discrimination, the corrected item–total correlation (CITC) results indicated that, except for EC-A4 (“regular weekly training”; CITC = 0.31), which was at the marginally acceptable level, all other items had CITC values greater than 0.40. This suggests that most items demonstrated satisfactory internal consistency with their respective dimensions.

Further “alpha if item deleted” analysis showed that removing EC-A4 would increase the Cronbach’s α coefficient of the F1 dimension from 0.89 to 0.90. However, given that this item effectively reflects the core practical characteristic of “training persistence” within the dance-specific context, and that its statistical indicator remained within the acceptable range, it was retained after considering content validity and theoretical representativeness.

It is noteworthy that the three items of the F5 (mind–body integration) dimension (EC-M1 to EC-M3) had CITC values of 0.48, 0.65, and 0.71, respectively. Although slightly lower than those of other dimensions, their discrimination indices were considered acceptable given the limited number of items in this dimension and its strong content specificity. The relatively lower CITC for EC-M1 (0.48) reflects the conceptual breadth of the item, which captures a general attentional disconnection state (“When I am focused, my mind and body feel completely disconnected”) rather than the specific bodily awareness loss targeted by EC-M3. This broader conceptual scope may reduce item-total correlation within the short three-item dimension, but the item was retained because it represents a core theoretical component of action-attention dissociation in dance performance that would be lost if deleted.

Overall, the items demonstrated satisfactory distributional properties and discrimination indices in the pilot phase. No abnormal items requiring forced deletion were identified, providing a reliable measurement foundation for subsequent exploratory and confirmatory factor analyses.

### 3.2 Exploratory factor analysis

The Kaiser–Meyer–Olkin (KMO) measure of sampling adequacy for the pilot sample (*N* = 136) was 0.827 (>0.80, excellent), and Bartlett’s test of sphericity was significant [χ^2^(351) = 3468.905, *p* < 0.001], indicating that the data were suitable for factor analysis.

Parallel analysis (based on 100 random data simulations) suggested the extraction of five factors, which together accounted for 58.42% of the total variance. In accordance with the multidimensional integrative framework of embodied cognition, a five-factor model was ultimately retained as the measurement structure.

The EFA showed that the items clustered meaningfully within their intended dimensions, with no substantial cross-loadings overall. The initial analysis included all pilot-phase items, including the original five items in the F5 dimension. Within F5, EC-M4 (“the body’s needs take priority”) and EC-M5 (“proud of my physical ability”) showed minor cross-loadings on F3 (0.32 and 0.35, respectively) and were judged to overlap conceptually with F3 items. To improve factor purity while preserving content validity, these two items were removed. The final EFA solution therefore retained 27 items: F1 (7 items), F2 (5 items), F3 (6 items), F4 (6 items), and F5 (3 items).

### 3.3 Confirmatory factor analysis

To determine the optimal structure of the Embodied Cognition Assessment Scale, the present study compared three competing models: a strictly unidimensional model (all 27 items loading on a single factor), a first-order correlated five-factor model, and a second-order single-factor model. The analyses were based on the 27-item data from the formal survey.

The unidimensional model showed markedly poorer fit: χ^2^(324) = 2156.43, *p* < 0.001, CFI = 0.612, TLI = 0.583, RMSEA = 0.142 (90% CI [0.137, 0.147]), SRMR = 0.112, AIC = 2248.43.

As shown in Table 2, the first-order five-factor model demonstrated the following fit indices: χ^2^(314) = 883.76, *p* < 0.001, CFI = 0.859, TLI = 0.842, RMSEA = 0.078 (90% CI [0.072, 0.084]), and SRMR = 0.057.

**TABLE 2:** Comparison of competing model fit indices for the Embodied Cognition Assessment Scale (ECAS) (*N* = 303).

Model	χ ^2^	df	χ ^2^/df	RMSEA (90% CI)	CFI	TLI	SRMR	AIC
Unidimensional model	2156.43	324	6.66	0.142 (0.137–0.147)	0.612	0.583	0.112	2248.43
First-order correlated five-factor model	883.76	314	2.82	0.078 (0.072–0.084)	0.859	0.842	0.057	1011.76
Second-order single-factor model	948.17	319	2.97	0.081 (0.075–0.087)	0.844	0.828	0.067	1066.17
Independence model	4385.66	351	12.5	0.195 (0.190–0.200)	–	–	–	–

According to Chen (2007), ΔCFI values greater than 0.01 indicate a significant difference between models. In the first-order model, an error covariance between F1 and F4 was added based on both theoretical justification and modification indices (MI) to improve local model fit. Theoretically, this covariance reflects the shared method variance between motor control items (e.g., “rapid adjustment to venue changes”) and cognitive flexibility items (e.g., “adjust quickly after plan failure”), both of which involve situational adaptation and executive control processes in dance performance contexts. The MI value for this path was 28.4, substantially exceeding the threshold of 3.84.

The second-order single-factor model showed relatively poorer fit: χ^2^(319) = 948.17, *p* < 0.001, CFI = 0.844, TLI = 0.828, RMSEA = 0.081 (90% CI [0.075, 0.087]), and SRMR = 0.067.

Model comparison indicated that the first-order model outperformed both alternative models across all fit indices. Relative to the unidimensional model, the improvement was substantial (ΔCFI = 0.247; ΔRMSEA = 0.064), demonstrating that the five-factor structure captures meaningful multidimensionality beyond a single general embodied cognition factor. Relative to the second-order model, the first-order model showed better fit (ΔCFI = 0.015 > 0.01; ΔRMSEA = 0.003). Furthermore, the AIC value of the first-order model (1011.76) was lower than that of the second-order model (1066.17), supporting the adoption of the first-order five-factor structure.

Although the CFI and TLI values of the first-order model were slightly below the conventional 0.90 threshold, RMSEA < 0.08 and SRMR < 0.08, together with χ^2^/df = 2.82 < 3.0, indicated that the overall model fit was within an acceptable range. Given that the present study included 27 observed variables and five correlated factors, the model was relatively complex, and CFI and TLI are known to be sensitive to model misspecification in complex measurement models (Goretzko et al., 2023; Xia and Yang, 2019). In addition, GFI = 0.819 ( > 0.80) and NFI = 0.798 (approaching 0.80) further supported adequate model fit.

The standardized factor loadings in the first-order model ranged from 0.60 to 0.87, all exceeding the recommended threshold of 0.60, indicating strong explanatory power of the observed variables on their respective latent constructs. Specifically, the loading ranges were as follows: F1 (motor control), 0.72–0.89; F2 (rigid response), 0.68–0.76; F3 (positive body self-image), 0.75–0.86; F4 (cognitive flexibility), 0.69–0.79; and F5 (mind–body integration), 0.60–0.70. All loadings were statistically significant at *p* < 0.001.

According to the recommendations of Goretzko et al. (2023), Xia and Yang (2019), in complex measurement models involving multiple factors and a relatively large number of items (k = 27), CFI and TLI are often more difficult to reach the strict 0.90 criterion.

**FIGURE 1:**
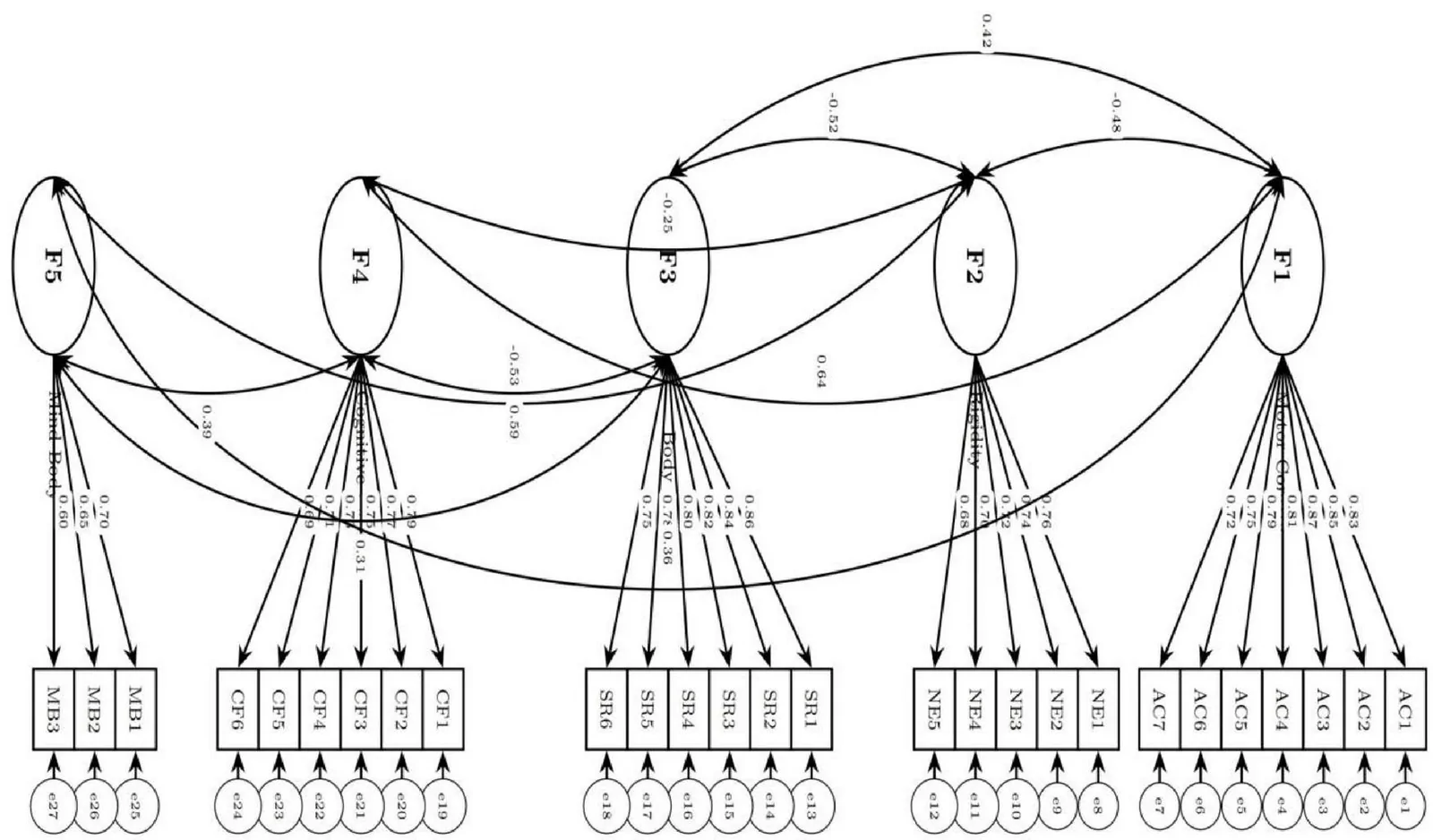
Confirmatory factor analysis model of the embodied cognition scale for dance students (ECAS-27). F1, Motor Control and Action Efficacy; F2, Rigid Response and Negative Experience (reverse-scored); F3, Positive Body Self-Image; F4, Cognitive Flexibility; F5, Mind–Body Integration (reverse-scored). AC, action control items; NE, negative experience items; SR, self-representation items; CF, cognitive flexibility items; MB, mind–body integration items. All standardized factor loadings were significant at *p* < 0.001. Model fit indices: χ^2^(314) = 883.76, CFI = 0.859, TLI = 0.842, RMSEA = 0.078 (90% CI [0.072, 0.084]), SRMR = 0.057.

Figure 1 presents the standardized path coefficients of the first-order five-factor CFA model of the ECAS-27. Results indicated that the standardized factor loadings of all items on their corresponding latent variables ranged from 0.60 to 0.87 (all *p* < .001), demonstrating strong explanatory power of the observed variables for their respective constructs.

Specifically, the seven items of F1 (Motor Control and Action Efficacy) had loadings ranging from 0.72 to 0.89; the five items of F2 (Rigid Response and Negative Experience) ranged from 0.68 to 0.76; the six items of F3 (Positive Body Self-Image) ranged from 0.75 to 0.86; the six items of F4 (Cognitive Flexibility) ranged from 0.69 to 0.79; and the three items of F5 (Mind–Body Integration) ranged from 0.60 to 0.70.

Inter-factor correlations (see latent variable paths in Figure 1) showed that F1 and F4 were moderately positively correlated (*r* = 0.64), consistent with the theoretically expected coupling between motor control and cognitive flexibility. F2 and F5 were moderately positively correlated (*r* = 0.53), reflecting the intrinsic association between rigidity and dissociative experience. The remaining correlations ranged from low to moderate (|r| = 0.25–0.52), supporting the discriminant validity of the five-factor structure. All error terms (e1–e27) were significant and correctly specified, with no improper estimates, indicating adequate model identification.

### 3.4 Measurement invariance testing

To examine the measurement invariance of the ECAS across subgroups, multi-group confirmatory factor analysis (MGCFA) was conducted across gender (male vs. female) and dance style groups. Following Chen (2007), changes of ΔCFI < 0.01 and ΔRMSEA < 0.015 were used as criteria for invariance. The configural models showed acceptable fit across comparisons, supporting a similar factor structure across groups. Relative to the configural models, the metric models yielded changes in CFI below 0.01, indicating equivalence of factor loadings. Subsequent tests of scalar invariance showed that although the chi-square difference tests were statistically significant, the changes in CFI remained within the recommended threshold, supporting intercept invariance. Taken together, these results suggest that the ECAS demonstrates an acceptable level of measurement invariance across the examined subgroups, allowing group comparisons to be interpreted with reasonable confidence.

### 3.5 Reliability analysis

The Cronbach’s α coefficients and composite reliability (CR) values for each dimension in the formal sample (*N* = 303) are presented in Table 3.

**TABLE 3:** Reliability indices of the Embodied Cognition Assessment Scale (*N* = 303).

Dimension	No. of items	Cronbach’s α	Standardized α	Composite reliability (CR)
F1 Motor Control and Action Efficacy	7	0.89	0.9	0.88
F2 Rigid Response and Negative Experience	5	0.76	0.76	0.89
F3 Positive Body Self-Image	6	0.9	0.9	0.91
F4 Cognitive Flexibility	6	0.8	0.8	0.86
F5 Mind–Body Integration (reverse-scored)	3	0.64	0.64	0.85

F5 (Mind–Body Integration, reverse-scored) is a short dimension consisting of three items. The results indicated satisfactory internal consistency across all five dimensions, with Cronbach’s α coefficients ranging from 0.76 to 0.90 and composite reliability (CR) values exceeding 0.80 for F1–F4, thereby demonstrating robust measurement properties for the majority of the scale.

Notably, F5 (Mind–Body Integration) presented a unique psychometric profile: although its Cronbach’s α (0.64) fell marginally below the conventional 0.70 threshold, its CR reached 0.85, well above the recommended cutoff. This apparent discrepancy reflects the dimension’s limited number of items (k = 3) and the use of reverse-scored indicators, conditions under which Cronbach’s α is known to underestimate reliability, whereas CR provides a more accurate estimate (Peterson and Kim, 2013; Cho and Kim, 2015). Given F5’s theoretical indispensability for capturing mind–body dissociation in dance training and its strong content representativeness, the dimension was retained based on its adequate CR and satisfactory average variance extracted (AVE = 0.65).

### 3.6 Validity analysis

Convergent validity: The average variance extracted (AVE) values for each dimension ranged from 0.50 to 0.65 (see Table 4). F4 (Cognitive Flexibility, AVE = 0.50) and F5 (Mind–Body Integration, AVE = 0.65) both reached or approximated the recommended threshold of 0.50. Combined with CR values above 0.80, convergent validity was considered acceptable. Notably, although F5 consisted of only three items, its AVE (0.65) and CR (0.85) demonstrated strong performance, indicating good convergent validity for this short dimension.

**TABLE 4:** Convergent and discriminant validity results.

Dimension	CR	AVE	√AVE	F1	F2	F3	F4	F5
F1	0.88	0.51	0.71	–	–	–	–	–
F2	0.89	0.62	0.79	0.48	–	–	–	–
F3	0.91	0.63	0.79	0.42	0.52	–	–	–
F4	0.86	0.5	0.71	0.64	0.25	0.59	–	–
F5	0.85	0.65	0.81	0.36	0.53	0.31	0.39	–

Diagonal elements (√AVE) represent the square root of the average variance extracted. Off-diagonal elements represent inter-factor correlations. Discriminant validity is supported when √AVE exceeds inter-construct correlations (Fornell and Larcker, 1981).

Discriminant validity: The Fornell–Larcker criterion indicated that the square roots of AVE for all dimensions (0.71–0.81) exceeded the corresponding inter-factor correlations (0.25–0.64). The heterotrait–monotrait ratio (HTMT) analysis further showed that all HTMT values were below the stringent threshold of 0.85 (range: 0.42–0.78). These results suggest that while the dimensions exhibited theoretically expected low-to-moderate correlations, they maintained adequate conceptual distinctiveness.

Criterion-related validity: To examine associations between the embodied cognition scale and external criterion variables, psychological resilience (Connor and Davidson, 2003). Connor-Davidson Resilience Scale, CD-RISC) and years of dance training were included as criterion measures. The total embodied cognition score was significantly positively correlated with psychological resilience (*r* = 0.52, *p* < 0.001). These correlations represent preliminary evidence for criterion-related validity, as the cross-sectional design precludes causal inference. The effect sizes range from small to medium (Cohen, 1988), with the total score-resilience correlation approaching a large effect. Future longitudinal or intervention studies are needed to establish predictive validity.

At the dimensional level, cognitive flexibility (F4) showed the strongest association with resilience (*r* = 0.48, *p* < 0.001), followed by positive body self-image (F3) (*r* = 0.45, *p* < 0.001). In addition, years of dance training were positively correlated with motor control (F1) (*r* = 0.31, *p* < 0.001) and negatively correlated with rigid response (F2) (*r* = −0.24, *p* < 0.001). These patterns are consistent with theoretical expectations and support the criterion-related validity of the embodied cognition scale.

In addition, to examine discriminant validity relative to existing embodied cognition measures, a subsample (*n* = 156) was randomly selected from the formal survey participants to complete two subscales of the Multidimensional Assessment of Interoceptive Awareness (MAIA), namely Noticing and Emotional Awareness. This subsample did not differ from the remaining participants in demographic characteristics (gender, grade, dance style, years of training; all *p*_s_ > 0.20). Results showed that ECAS Cognitive Flexibility (F4) was moderately correlated with MAIA Noticing (*r* = 0.42, *p* < 0.01), but this association was significantly lower than its internal correlation with F1 (*r* = 0.64), supporting convergent validity. Importantly, ECAS Motor Control (F1) demonstrated relatively low correlations with MAIA dimensions (*r* = 0.18–0.25), suggesting that the “action-based embodied cognition” assessed by ECAS differs theoretically from the “resting-state interoceptive awareness” measured by MAIA. These findings support the specificity of ECAS in capturing “doing through the body” in dance contexts, rather than redundantly measuring general bodily awareness.

## 4 Discussion

### 4.1 Theoretical interpretation and measurement implications of the five-factor structure

Model comparison results indicated that simplifying embodied cognition into a single higher-order factor led to a meaningful loss of structural information (ΔCFI = 0.015 > 0.01). The comparison with a strictly unidimensional model further demonstrated that collapsing all 27 items into a single factor produced markedly deficient fit (CFI = 0.612, RMSEA = 0.142), confirming that the five-factor structure captures meaningful multidimensionality beyond a general embodied cognition factor. In contrast, the first-order five-factor structure provided a better representation of the data. This finding aligns with embodied/grounded cognition perspectives emphasizing that cognition is rooted in body–environment interaction and organized into distinguishable yet coordinated functional modules serving situated action (Barsalou, 2020).

Physical education objectives have long been conceptualized as encompassing relatively distinct domains of cognitive, affective, and psychomotor development, providing a structural reference for domain-specific development in dance training contexts.

Specifically, the strong correlation between Motor Control and Action Efficacy (F1) and Cognitive Flexibility (F4) (*r* = 0.64) suggests a stable “action–cognition coupling” in dance training. Theoretical and review literature consistently emphasizes that motor systems are not merely output channels of cognition but play a constitutive role in understanding, reasoning, and learning (Glenberg, 2015; Barsalou, 2020). Consistent with this view, numerous neuroimaging studies and reviews have reported associations between dance expertise and functional/structural brain differences, particularly involving the action observation–execution network, premotor cortex, and parietal systems (Calvo-Merino et al., 2005; Vander Elst et al., 2023). Thus, the significant association between F1 and F4 likely reflects cross-system coupling induced by training rather than lack of discriminability between dimensions.

Compared with general interoceptive/body awareness scales, the present instrument demonstrates “motor-experience dominance,” with F1 and F3 (Positive Body Self-Image) occupying relatively central explanatory roles in the structure. This distinction is consistent with the measurement positioning of MAIA, which emphasizes multidimensional self-reported interoceptive awareness but was primarily developed for body-awareness–oriented therapeutic populations and general samples, potentially underrepresenting the dynamic motor control and real-time regulatory features of high-skill physical practice (Mehling et al., 2012).

Methodologically, the present findings support the necessity of context-specific measurement: direct transplantation of clinical/general population instruments may underestimate key differences related to action efficacy, motor regulation, and executive control in dance training.

Importantly, the inclusion of F5 (Mind–Body Integration, reverse-scored to reflect disconnection) enhances sensitivity to the complex phenotype of mind–body dissociation/integration. Empirical studies have shown that professional dancers exhibit higher interoceptive accuracy (e.g., heartbeat detection tasks), suggesting that long-term training may alter bodily signal processing and attentional allocation (Christensen et al., 2018). However, interoceptive accuracy is not equivalent to mind–body integration, underscoring the necessity of distinguishing awareness capacity from integrative state at the measurement level.

It should also be noted that reverse-worded items may introduce method effects and distort structural fit, particularly in short dimensions. The three F5 items (EC-M1 to EC-M3) are all reverse-scored, measuring dissociative experience rather than direct integration. This uniform item directionality raises the possibility of a method factor distinct from the substantive construct, as systematic response patterns to negatively worded items may inflate correlations among F5 indicators independently of true mind–body integration levels (İlhan, 2024). Therefore, item directionality and potential method factor control for F5 should be prioritized in future research.

### 4.2 Comparison with existing instruments

Compared with existing embodied cognition and body awareness instruments, ECAS demonstrates two structural distinctions.

First, whereas MAIA and similar scales emphasize dimensionalized evaluation of “attending to the body” or “awareness of the body,” ECAS places greater emphasis on “motor control–action efficacy” and “cognitive flexibility” within dance contexts (Mehling et al., 2012; Barsalou, 2020). This difference corresponds to the real-time predictive and adaptive demands of dance training in rhythm, spatial coordination, and movement sequencing, and aligns with neuroscientific findings highlighting interactions between motor skill acquisition and cognitive control systems (Calvo-Merino et al., 2005; Vander Elst et al., 2023).

Second, ECAS incorporates “Rigid Response and Negative Experience” (F2), a dimension often underemphasized in general embodied cognition measures but highly relevant in high-pressure training contexts. Research on professional dance samples has documented elevated risk profiles for body-related self-evaluative anxiety (e.g., social physique anxiety, body dissatisfaction) (Quested and Duda, 2009). The significant negative correlation between F2 and psychological resilience (*r* = −0.41) indicates that embodied cognition encompasses not only positive bodily experiences but also patterns of responding to bodily limitation, pain, and stress. This aligns with evidence suggesting that positive and negative body image dimensions may coexist and serve distinct functions (Tiggemann, 2015; Linardon et al., 2022).

Furthermore, the five-factor structure avoids reducing bodily experience to attitudinal evaluation by capturing situational adaptation and attention–action synchrony through F4 (Cognitive Flexibility) and F5 (Mind–Body Integration). Dance intervention research has also reported associations between dance training and improvements in working memory, inhibitory control, and cognitive flexibility, providing additional educational interpretation for F4 (Wang et al., 2025).

### 4.3 Implications for dance education and psychological intervention

The five-factor structure of ECAS provides an operational framework for “diagnosis–feedback–adjustment” in dance education. Skill learning and physical education research emphasize differentiated development across cognitive, affective, and psychomotor domains, supporting multidimensional scoring approaches in instructional design.

Within this framework, individuals scoring lower on F4 may benefit from improvisation and task-switching training to enhance executive function, whereas those scoring lower on F3 may benefit from feedback strategies promoting positive body image. Substantial evidence from reviews, meta-analyses, and cross-cultural data supports stable positive associations between positive body image and psychological adaptation indicators (Tiggemann, 2015; Linardon et al., 2022; Swami et al., 2023).

In terms of intervention formats, embodied mindfulness and psychological training approaches increasingly adopt embedded designs that align with training load and contextual constraints. Scoping reviews suggest that dance-related mindfulness and mind–body interventions may influence body awareness, emotion regulation, and stress indicators, offering potential pathways for enhancing F5 (Mind–Body Integration) (Zafeiroudi et al., 2025).

Given that F2 may indicate risk patterns of negative experience and rigidity, the scale may also assist in identifying subgroups requiring prioritized psychological support, thereby aligning dance education practices with evidence-based screening principles.

### 4.4 Limitations and future directions

Although RMSEA and SRMR of the first-order five-factor CFA model were within acceptable ranges, CFI and TLI were slightly below the conventional 0.90 threshold. It is increasingly recognized that fixed cutoff criteria should not be applied mechanically: fit indices are influenced by model complexity, estimation method, and data characteristics, and rigid application of single thresholds may lead to misinterpretation (Xia and Yang, 2019; Goretzko et al., 2023; Groskurth et al., 2024). Future research may adopt model-tailored fit evaluation strategies and replicate structural stability in independent samples.

Second, the post hoc addition of an error covariance between F1 and F4 based on modification indices in the same sample used for final model reporting should be explicitly acknowledged as an exploratory adjustment. While this covariance was theoretically justified by the shared situational adaptation content between motor control and cognitive flexibility items, future studies should cross-validate this path in independent samples to confirm its stability. We recommend that subsequent research either replicate this covariance structure or test alternative model specifications (e.g., correlated uniqueness models) to rule out sample-specific capitalization on chance.

Third, F5 consisted of only three reverse-worded items, with Cronbach’s α at a marginal level. Reverse-worded strategies may introduce method factor effects and spurious dimensionality, particularly in short scales. We explicitly acknowledge this risk: the uniform reverse-wording of all three F5 items creates a potential method factor that may confound the measurement of mind–body integration. Future studies should (a) expand F5 with at least one positively worded item (e.g., “I feel completely connected between my mind and body during performance”) to balance item directionality; (b) explicitly test a bifactor model with a method factor for reverse-worded items to quantify method variance; and (c) consider item response theory (IRT) analysis to examine differential item functioning between positively and negatively worded indicators (İlhan, 2024). The psychometric consequences of reverse-worded items have been systematically discussed in recent literature (İlhan, 2024).

Fourth, the present sample was recruited via convenience sampling from a single provincial university in China, predominantly comprising undergraduate students in Chinese classical dance and folk dance specializations. This sampling strategy limits generalizability to professional dancers, international dance academies, and other dance genres (e.g., ballet, modern dance, street dance) that may exhibit distinct embodied cognition profiles. Future research should replicate the ECAS in professional dance companies, cross-cultural samples, and diverse dance genres to establish broader applicability. Additionally, the underrepresentation of male dancers (26.7%) and competitive dance students (24.1%) may restrict the scale’s validity in male-dominated or competitive dance populations.

Finally, although the two-stage EFA–CFA design reduced sample-specific bias, generalizability requires further confirmation through cross-group measurement invariance testing. The theoretical and procedural requirements of measurement invariance are well-established in methodological literature (Meredith and Teresi, 2006), and ΔCFI-based criteria are widely used to evaluate group equivalence (Chen, 2007). When invariance risks exist, recent methodological work suggests integrating mixture or multilevel SEM strategies to improve comparative validity (Zhao et al., 2025).

## 5 Conclusion

The present study developed and validated a 27-item, five-factor Embodied Cognition Assessment Scale (ECAS) for dance majors. Results support the multidimensional organizational perspective of embodied/grounded cognition, emphasizing distinguishable yet coordinated functional modules linking cognition and action systems.

The scale structure highlights key dimensions in dance training contexts, including motor control, cognitive regulation, and mind–body integration, and aligns theoretically with evidence of dance-related functional and structural brain differences.

At the same time, the dimensional design maintains dialogue with interoceptive measurement traditions: while evidence indicates enhanced interoceptive accuracy among dancers, measurement must distinguish between awareness and integrative mechanisms.

Future research should examine measurement invariance in broader samples and adopt more robust fit evaluation and method factor control strategies to enhance cross-context applicability.

## Data Availability

The datasets presented in this study can be found in online repositories. The names of the repository/repositories and accession number(s) can be found in the article/Supplementary material.

## Supplementary material

Supplementary Table 1 Detailed expert review ratings for the 40 initial ECAS items.

Supplementary Table 2 Measurement invariance testing of the ECAS-27 across gender (male vs. female) and dance style (classical dance vs. modern dance) groups.
