# Supplementary material for: Development and psychometric validation of a multidimensional measure of embodied cognition in dance training
Source: Front Psychol. 2026 Aug 7;17:1861862. doi: 10.3389/fpsyg.2026.1861862 (PMC13493231; doi:10.3389/fpsyg.2026.1861862)
Supplement: Supplementary file 1 [file Supplementary_file_1.docx]

Supplementary Material

# 1 Supplementary Data

Embodied Cognition Assessment Scale (ECAS-27)

Instructions: Please read each statement carefully and indicate how much you agree with it based on your experiences in dance training. Use the following scale: 1 = Strongly Disagree, 2 = Disagree, 3 = Neutral, 4 = Agree, 5 = Strongly Agree.

F1. Motor Control and Action Efficacy (7 items)

I have good coordination of my hands and limbs during movement.

I can control the force, speed, and direction of my movements precisely.

I can stably master new movements after a brief period of practice.

I maintain regular weekly training to consolidate my motor skills.

I can persist in training despite physical fatigue.

I can maintain endurance during moderate-intensity dance activities.

I can rapidly adjust my movements when the venue or conditions change.

F2. Rigid Response and Negative Experience (5 items) [Reverse-scored]

8. I feel anxiety or fear about my body's performance in dance.

9. I am concerned about appearance pressure from teachers or peers.

10. I panic when unexpected situations occur during performance.

11. I give up easily when dance activity becomes difficult.

12. I have difficulty adapting to unexpected changes in choreography.

F3. Positive Body Self-Image (6 items)

13. I feel satisfied with my body shape.

14. I feel satisfied with my movement quality and appearance.

15. I feel proud of my body's capabilities.

16. My body brings me joy during dance.

17. I feel my body is flexible and adaptable.

18. My whole body feels powerful when I dance.

F4. Cognitive Flexibility (6 items)

19. I can generate multiple options before making decisions in dance.

20. I verify information sources before accepting new movement techniques.

21. I can incorporate others' perspectives when learning choreography.

22. I weigh pros and cons before making decisions in improvisation.

23. I can adjust quickly after a plan or movement fails.

24. I can view dance issues from different angles.

F5. Mind-Body Integration (3 items) [Reverse-scored]

25. When I am focused, my mind and body feel completely disconnected.

26. During performance, I feel like my body is moving on its own, separate from my mind.

27. I often lose awareness of my body's present needs during training.

Scoring Notes:

All F2 and F5 items are reverse-scored: Raw score 1→5, 2→4, 3→3, 4→2, 5→1

After reverse-scoring, higher scores on all dimensions indicate higher levels of the construct

Dimension scores = mean of items within each dimension. Total ECAS score = mean of all 27 items (after reverse-scoring)
